# Glycolysis Rate-Limiting Enzymes: Novel Potential Regulators of Rheumatoid Arthritis Pathogenesis

**DOI:** 10.3389/fimmu.2021.779787

**Published:** 2021-11-24

**Authors:** Jianlin Zuo, Jinshuo Tang, Meng Lu, Zhongsheng Zhou, Yang Li, Hao Tian, Enbo Liu, Baoying Gao, Te Liu, Pu Shao

**Affiliations:** ^1^ Department of Orthopeadics, China-Japan Union Hospital of Jilin University, Changchun, China; ^2^ Department of Nursing, The First Bethune Hospital of Jilin University, Changchun, China; ^3^ Scientific Research Center, China-Japan Union Hospital of Jilin University, Changchun, China; ^4^ Department of Cardiology, China-Japan Union Hospital of Jilin University, Changchun, China

**Keywords:** glycolysis, rate-limiting enzymes, rheumatoid arthritis, RA, pathogenesis

## Abstract

Rheumatoid arthritis (RA) is a classic autoimmune disease characterized by uncontrolled synovial proliferation, pannus formation, cartilage injury, and bone destruction. The specific pathogenesis of RA, a chronic inflammatory disease, remains unclear. However, both key glycolysis rate-limiting enzymes, hexokinase-II (HK-II), phosphofructokinase-1 (PFK-1), and pyruvate kinase M2 (PKM2), as well as indirect rate-limiting enzymes, 6-phosphofructo-2-kinase/fructose-2,6-bisphosphatase 3 (PFKFB3), are thought to participate in the pathogenesis of RA. In here, we review the latest literature on the pathogenesis of RA, introduce the pathophysiological characteristics of HK-II, PFK-1/PFKFB3, and PKM2 and their expression characteristics in this autoimmune disease, and systematically assess the association between the glycolytic rate-limiting enzymes and RA from a molecular level. Moreover, we highlight HK-II, PFK-1/PFKFB3, and PKM2 as potential targets for the clinical treatment of RA. There is great potential to develop new anti-rheumatic therapies through safe inhibition or overexpression of glycolysis rate-limiting enzymes.

## Introduction

Rheumatoid arthritis (RA) is one of the most prevalent chronic inflammatory diseases. It involves chiefly the joints, but can present extra-articular manifestations, such as rheumatoid nodules, pulmonary involvement, vasculitis, and systemic comorbidities (1). RA’s prevalence has remained relatively stable in many populations (ranging from 0.5% to 1%) and it is at least twice more common in women than in men. The disease can occur at any moment, but the peak incidence arrives at approximately 50 years of age (2, 3). RA is mainly characterized by an inflammatory infiltration with abnormal vascular proliferation in the synovial membrane and pannus formation; recurrent attacks to the joints lead to cartilage destruction and bone erosion, eventually leading to deformation of the affected joint and even complete loss of its motor function (4). The immune cells in RA share an altered proliferative capacity that is evident in the joint-resident cells that form the synovial pannus (5). Synovial proliferation, neoangiogenesis, and leukocyte extravasation transform the normal noncellular synovium into an invasive tumor-like pannus (6). Pannus formation is one of the driving pathological processes leading to RA joint erosion (7). Energetic and biosynthetic precursors are on high demand during biomass construction, implying that metabolic control is fundamental during the pathogenesis of RA (5).

**Figure 1:**
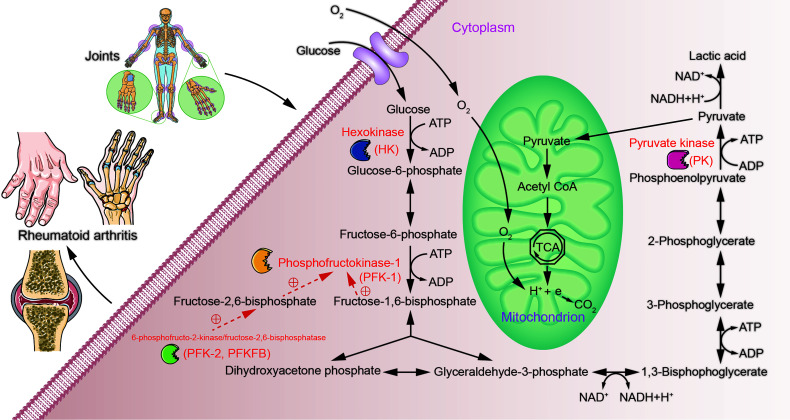
Possible role of glucose metabolism during RA. Over- or under-expression of glycolysis rate-limiting enzymes may contribute to metabolic differences in the tissues of patients with RA through aberrant regulation of glycolytic pathways.

Glucose metabolism involves a 10-step cytoplasmic reaction called glycolysis, or more formally, the glycolytic pathway. This 10-step reaction converts one molecule of glucose into two molecules of 3-carbon pyruvate. Glycolysis also creates two molecules of ATP and reduces two NAD+ molecules to NADH, these two types molecules are metabolic fuels that drive other biological reactions (8). Under aerobic conditions, the pyruvate in mitochondria can be used during aerobic respiration *via* the tricarboxylic acid cycle (TAC) to produce more ATP for the cell (9). Under low partial oxygen pressure, pyruvate is fermented by lactate dehydrogenase (LDH) in the cytoplasm into lactate while converting NADH to NAD+ (10). However, in the presence of sufficient oxygen and functional mitochondria, some cells display an enhanced and accelerated metabolic conversion of glucose into lactate, a phenomenon named the Warburg effect (11, 12), which can also be referred to as aerobic glycolysis (13). Chronic inflammatory disorders, such as rheumatosis, require a good deal of energy supplied and distributed to the activated immune system (14). RA is a classical rheumatic disease with high metabolic demands (15), but its multiple pathological alterations maintain the affected tissues in a hypoxic state (16, 17). Therefore, complex alterations must be present in tissue and cellular metabolic pathways. Studies have shown that glycolysis, an important process during glucose metabolism, plays a significant role in the pathogenesis of RA (18, 19). Herein, we systematically describe the association between glycolysis and RA from the perspective of glucose metabolism and the key rate-limiting enzymes in the glycolytic pathway (**Figure 1**).

## Glycolysis and RA

During RA, hypoxia alters the cellular bioenergetics by inducing mitochondrial dysfunction and promoting a switch to the glycolytic pathway that leads to abnormal angiogenesis, cellular invasion, and pannus formation (20). Angiogenesis is a result of the hypoxic state and is a prominent feature of rheumatoid synovitis (21). Although some neovascularization delivers oxygen to the increased inflammatory cell mass, the neovascular network is dysfunctional and unable to restore tissue oxygen homeostasis, that’s why the joints in RA remain in a rock-ribbed hypoxic environment (22). The synovial fluid oxygen tension of the knee in patients with RA is significantly lower than that in patients with osteoarthritis (OA) with synovial hyperplasia (23). RA synovial fluid presents significant elevations in lactate and decreases in glucose concentrations as a consequence of the high synovial cell metabolic demands (24–26). By applying magnetic resonance spectroscopy (MRS), it was found that anaerobic metabolism becomes more pronounced with the progressive increase in the degree of inflammation and vascularity of the RA synovium (27). Magnetic resonance spectroscopic imaging of hyperpolarized [1-^13^C]-pyruvate metabolism has also demonstrated an increase in the lactate-to-pyruvate ratio within hind paws of the complete Freund’s adjuvant (CFA) RA arthritis model. This soar indicates enhanced glycolysis and an increased lactate concentration associated with acidosis, as observed in the synovial fluid of patients with RA (28). In addition, roles for glycolysis, fatty acid, and amino acid metabolism, and other related pathways (TCA and urea cycle) in RA have been revealed by gas chromatography-mass spectrometry (GC-MS) experiments showing decreased levels of amino acids and glucose and increased levels of fatty acids and cholesterol in sera of patients with RA (29). Metabolic changes of glucose, phospholipids, and bioactive lipids (such as sphingosine-1-phosphate and lysophosphatidic acid) are needed during activation of fibroblast-like synoviocytes (FLSs) and may contribute to initiation of immune responses or abnormal immune responses that trigger RA and contribute strongly to joint destruction (30).

In RA, FLSs, which form the articular lining, exert a significant part in pathological processes and are epigenetically imprinted with an aggressive phenotype (31). FLS are the most common cell type at the pannus-cartilage junction, triggering joint destruction through the production of cytokines, chemokines and matrix degradation molecules as well as migration and invasion (30, 32). These pathological features are inevitably linked to tumor-like phenotypes of FLSs. The rate of proton efflux, proton (H+) production rate (PPR), and the mitochondrial respiration rate, oxygen consumption rate (OCR), were measured in RA and OA FLSs cell lines using a Seahorse XF analyzer, and the ratio of PPR to OCR represents an estimate of the relative balance between glycolysis and oxidative phosphorylation. RA FLSs have a higher PPR : OCR ratio than OA FLSs, suggesting that the balance between glycolysis and oxidative phosphorylation shifts to the glycolytic pathway in RA FLSs compared to the balance in human OA FLSs (33). Activation of FLSs by CD4 T cells increases their energy demands. The cells shift rapidly from oxidative phosphorylation to anaerobic glycolysis to support abnormal behaviors (proliferation, invasion, and conglutination) and to accelerate cytokine release (34). Substantial flux control is performed in four steps: glucose import, hexokinase (HK), phosphofructokinase (PFK), and lactate export. These four flux control steps are specifically upregulated by the Ras oncogene: optogenetic Ras activation rapidly induces transcription of isozymes that catalyze these four steps, thereby enhancing glycolysis (35). T cells from patients with RA express significantly high levels of K-RAS, and present a hyperactivated Ras/MEK/ERK pathway (36, 37). Multiple Ras proteins are expressed in both synovial tissue (ST) and cultured FLSs, and Ras protein expression and activation changes lead to the pathological phenotype of FLSs in RA (38). Additionally, IgG immune complexes sensitize human monocytes for overactive inflammation through transcriptomic and epigenetic reprogramming in RA (39). This suggests that RA specific autoantibodies can train monocytes in inflammatory lesions as early as the asymptomatic stage, when enhanced glycolysis in RA diseased region is already occurring (40). An alteration of energy metabolism is clear from the initial stages of RA; glucose, the main energy supplier of the body, is preferentially utilized. The associations between altered glucose metabolism, glycolysis, and RA should be understood first.

## Glycolysis Rate-Limiting Enzymes and RA

Studies have implicated numerous energy metabolic enzymes such as glucose phosphate isomerase (41), α-enolase (ENO1) (42, 43), aldolase (25), and triosephosphate isomerase in RA glycolysis mechanisms (44). However, most of the 10 reactions of the glycolytic pathway are reversible, and the direction and rate of these reactions are controlled by the concentrations of their substrates and products. Changes in the activity of the enzymes that catalyze these reversible reactions do not determine the direction of the reactions (45). Instead, the control of the flow of glycolysis depends primarily on the activity of three key rate-limiting enzymes, hexokinases (HKs), phosphofructokinase-1 (PFK-1), and pyruvate kinases (PKs) (46, 47). These three key enzymes are regulated by irreversible reactions within the cell and the rate of these reactions is slow (45). By regulating glucose metabolism, glycolytic rate-limiting enzymes become crucial regulators of the RA pathogenesis.

HKs are the first rate-limiting enzymes in the glycolytic pathway. Glucose transported into the cell *via* glucose transporter (GLUT) is phosphorylated by HKs to glucose-6-phosphate (G-6-P) (48), a process that is efficient and irreversible, and it has evident flux control (35, 49). There exist four isoforms of HKs in mammalian tissues, HK-I, HK-II, HK-III and HK-IV, which differ in their major distribution in various tissues of the body (50). The ubiquitous HK-I isoform seems to be constitutively expressed in most tissues. HK-II is a major regulated isoform in many cell types and is widely expressed in insulin-sensitive tissues such as muscle and adipose, which are the bulk of peripheral glucose utilization. HK-II is also widely expressed in many highly glycolytic cancers (51). For instance, overexpression of HK-II promotes cell migration and invasion *via* the FAK/ERK1/2/MMP-9 pathway and enhances stemness properties *via* the FAK/ERK1/2/NANOG/SOX9 cascade. HK-II abrogation inhibits tumor growth and spread *in vivo (* 52).

PFK-1 is the second key rate-limiting enzyme of glycolysis. PFK-1 is a tetrameric protein with three genes encoding human isoforms: PFK-M (muscle), PFK-L (liver), and PFK-P (platelet) (53). PFK-1 phosphorylates fructose 6-phosphate (F-6-P) into fructose 1,6-bisphosphate (F-1,6-BP), in another irreversible reaction that is far from equilibrium and constitutes a critical control point for the regulation of glycolytic flux (54). The rate of PFK-1 is influenced by a variety of allosteric effectors in the cytoplasm. The most potent allosteric effector of PFK-1 is fructose 2,6-bisphosphate (F-2,6-BP) (55). The interplay between F-2,6-BP levels, the enzymes that produce and degrade it, and PFK-1 activity have important implications for different aspects of cellular metabolism as well as for systemic metabolic conditions (56). F-2,6-BP levels are closely related to 6-phosphofructo-2-kinase/fructose-2,6-bisphosphatase (PFK-2, PFKFB), a bifunctional enzyme containing a phosphatase domain that can consume F-2,6-BP (35), and is responsible for the synthesis and degradation of F-2,6-BP. PFKFB controls the glycolytic flux by limiting the intracellular concentration of F-2,6-BP (55, 57). The human body has four isozymes of PFKFB, namely PFKFB1, PFKFB2, PFKFB3 and PFKFB4, which display tissue-specific expression patterns and different kinase-to-phosphatase activities. All four isozymes are induced by hypoxia *in vivo*, however, the hypoxia responsiveness varies in different organs (58).

PKs catalyze the tenth and final reaction of glycolysis: the irreversible conversion of ADP and phosphoenolpyruvate to ATP and pyruvate, both of which are essential for cellular metabolism (59). The PK family contains four isoforms encoded by two distinct PK genes (60). PKR is the only isoform expressed in erythrocytes; PKL is the dominant isoform in liver; and PKM1 is the dominant isoform in differentiated skeletal muscle, heart, and brain; unlike all other isoforms, PKM2 is present in numerous differentiated adult tissues (61). PKM1 and PKM2 are encoded by alternative splicing of the PKM gene, which is significantly regulated by three heterogeneous nuclear ribonucleoproteins (hnRNPA1, A2, and I) dependent on c-Myc (60). PKM2, a crucial rate-limiting enzyme of glycolysis, is normally overexpressed in proliferating and tumor cells, it regulates glycolysis and the Warburg effect (62). PKM2 is associated with some cancers and contributes to the direction of the glycolytic pathway into fermentation and the lactate formation (63). PKM2 contributes to TLR-mediated inflammation and autoimmunity and may be a promising target for controlling inflammation and autoimmunity (64). In addition, PKM2 is required for Th1 and Th17 differentiation *in vitro* and *in vivo*. PKM2 also represents a therapeutic target for T cell-dependent autoimmune diseases (65).

CD4+ T lymphocytes play a role in the pathogenesis of RA, forming a regulatory and functionally multitudinous population within the immune system (66). CD4+ T secretory factors induce a metabolic shift in FLSs from an oxidative metabolism to a more glycolytic phenotype. In primary RA FLSs activated by CD4 T cell-conditioned medium (CM), the mRNA expression levels of GLUT1, GLUT3, HK-II, PFKFB3, LDHA, and GSK3A are increased (34). Oncostatin M (OSM) is highly expressed in RA joints (67). OSM is a member of the IL-6 subfamily produced by inflammatory cells and some tumor cells and shares a common receptor signaling subunit (gp-130) with IL-6-type cytokines (68–70). OSM regulates metabolic reprogramming in RA FLSs in conjunction with TNFα, and it boosts mRNA expression of GLUT1, HK-II, PFKFB3, PKM2, and LDH through STAT3 phosphorylation (67). Many glycolytic pathway enzymes (including HK-III, PFK, PKM2, and ENO) have been shown to be upregulated in plate-bound human IgG-trained monocytes compared to their levels in blank controls (40). Upon encountering ICOSL+ B cells, activated effector memory TH cells from patients with RA spontaneously differentiate into inflammatory TH subsets. ICOSL-induced glucose uptake is involved in inflammatory TH polarization by B cells, and glycolysis is significantly upregulated in T cells polarized by B cells. Key enzymes of the glycolytic pathway (HK, PFKL, PFKFB3, and PKM) get significantly overexpressed in TH cells that have interacted with B cells (71). Identifying the exact role, such as inflammation, invasion, and proliferation, of the key rate-limiting enzymes of glycolysis pathway in RA, is crucial for understanding the relationship between energy metabolism and the occurrence and development of RA, and it is essential to completely elucidate the pathogenesis of RA.

## HK-II

Immunohistochemistry (IHC) methods have shown higher HK-I, HK-II, and HK-IV expression levels in the STs of patients with RA than in those of patients with OA (72). A quantitative real-time polymerase chain reaction (RT-qPCR) comparison of the expression levels of glycolytic related genes in RA FLSs and OA FLSs revealed that the mRNA levels of HK-II are significantly higher in the RA FLSs than in the OA FLSs (73). Moreover, HK-II is more differentially expressed in RA STs than the other HK isoforms (72), being very dominant in both the lining and sublining (18). The expression of GLUT1 mRNA is higher in RA FLSs than in OA FLSs, and the level is closely correlated with that of HK-II (33). Higher expression of metabolic enzymes and proteins associated with glycolysis, HK-II, GLUT1, LDHA, monocarboxylate lactate transporter 4 and HIF-1α, was also observed in RA synovial membrane CD8+ T cells (74). Primary bovine synovial cell cultures treated with different lipopolysaccharide (LPS) concentrations for 12 hours showed enhanced HK-II mRNA expression (starting at 100 ng/ml of LPS) increasing in a dose-dependent manner; the expression boost was maximal at 1 μg/ml of LPS (75). Double immunohistochemistry can be used to determine whether FLSs express HK-II in the RA synovium, these cells can be identified by their positivity for vimentin, vascular cell adhesion protein 1 (VCAM-1), or podoplanin (PDPN) (76–78). HK-II positive staining co-localized with all the diverse FLS markers in the lining (18). Stimulation with activated-Th cells-conditioned media (ThCM) results in a significant increase in HK-II mRNA expression in the RA synovial fibroblasts (SFs) compared with the levels under unstimulated conditions (79). Adjuvant arthritis (AA) is a commonly used animal model for human RA that can be used to score synovial proliferation, seep inflammatory cells, and cartilage destruction at different stages of the progressive disease (PD). The severity of pathological changes increases gradually with PD, while the expression of HK-II in ST was detected, and HK-II expression soared significantly with PD (80). Moreover, OSM significantly induces angiogenic networks formation, adhesion, and invasion mechanisms that are accompanied by changes in the bioenergetic profile of cells, in which OSM significantly increases the extracellular acidification rate (ECAR)/OCR ratio of primary RA FLSs (favoring glycolysis) and it induces the expressions of GLUT1 and of the key glycolytic rate-limiting enzymes HK-II and PFKFB3 (67).

Intra-articular injection of adenovirus carrying murine HK-II (ad-mHK-II) in the knee of healthy mice significantly increases the synovial lining thickness, and promotes FLS activation and proliferation. Overexpression of HK-II in the synovium of healthy mice transforms thin linings into hypertrophic synovial membranes, which also enhances FLS migration to the cartilage. HK-II causes healthy natural synovial linings to become hypertrophic with upregulated α-sma and matrix metalloproteinase-3 (MMP-3) expressions (18). K/BxN arthritis mice are a spontaneous model driven by T cell receptor transgenic CD4+ T cells from the KRN strain that are activated by G-6-P isomerase peptides presented by the H-2g7 allele from the NOD strain (81). HK-II is highly expressed in the synovial lining after K/BxN serum transfer arthritis (18). HK-II plays a pivotal role converting glucose into subsequent products of the glycolytic pathway and enhancing the cellular metabolic activity. Fortified HK-II activity is associated with major pathways of inflammation, angiogenesis, migration, invasion, and cell survival in FLSs (82). HK-II also increases the extracellular lactate production (83). Both HK-I and HK-II similarly increase the extracellular lactate levels, but only HK-II overexpression triggers an aggressive FLS phenotype, this suggests the presence of a glycolytic-independent mechanism (18).

Silencing of HK-I/II, siHK-I/II, or the use of an HK inhibitor like lonidamine (LND) decreases cell viability and reduces the production of proinflammatory cytokine and chemokines, IL-6, IL-8, CXCL9, CXCL10, and CXCL11 (72). Under normoxic conditions, Hif-1α knockdown reduces glycolytic metabolism and induces apoptosis in SFs (84). LND also induces apoptosis in RASF (72), but HK-I- or HK-II-silencing does not alter cell viability or shape (18). Inhibition of HK-II inhibits synovial cell activation *via* the AMPK/NF-кB pathway to improve arthritic symptoms in AA (80). 2-DG is a synthetic glucalogue in which the 2-hydroxyl group has been replaced by a hydrogen (85). 2-DG inhibits phosphorylation of other available sugars, such as glucose, and may act as a noncompetitive antagonist of HKs to restrain the cellular glycolytic activity and regulate glycolysis, leading to a decrease in intracellular ATP production (86, 87). 2-DG (200 mg/kg) treatment has been shown to increase the expression of p-AMPK proteins and to decrease the expression of p-p65 and p-IκBα proteins, suggesting that 2-DG may activate the AMPK pathway (80). RA FLSs pretreated with 2-DG and then cultured in the presence of platelet-derived growth factor-BB (PDGF-BB) for 4 days, exhibited a significantly diminished cell proliferation rate as measured by an MTT assay; the cellular levels of cell migration, IL-6, and MMP-3 were also significantly reduced (88). 2-DG treatment reverses LPS-induced increases in GLUT1 and HK-II mRNA expression. The compound reduces the dependence of synovial cells on the glycolytic pathway for ATP production by enhancing the contribution of mitochondrial respiration (MR) to ATP production and inhibiting LPS-stimulated lactate production (75). Human monocyte-derived dendritic cells (moDC) stimulated in the presence of 2-DG exhibit an impaired glycolysis due to HK activity inhibition. 2-DG prevents the cytokine production induced by individual Toll-like receptor (TLR) stimulation. Additionally, 2-DG also strongly restrains cytokine production after co-stimulation with complexed IgG and Pam3CSK4 (89). HK-II^Col1^ mice (HK-II deleted in HK-II^Col1^ joint FLSs) present a significant reduction in arthritis severity, and in bone and cartilage damages as compared to manifestations in control mice (18).

3-Bromopyruvate (BrPA), a specific HK-II inhibitor, significantly reduces the arthritis and the histological scores in the SKG mouse model (a genetic model with many RA features) while significantly increasing the number of regulatory T cells (Treg). *In vitro*, BrPA promotes differentiation of Treg cells, suppresses interleukin-17-producing T cells (Th17), and inhibited the activation of dendritic cells (90). Treatment with LND in a type II collagen-induced arthritis (CIA) in DBA-/1 mouse model reduces the production of antibodies against IgG1, IgG2a, and IgG2b, thereby reducing articular inflammation and destruction. LND delays illness in an animal model of RA, and it may get developed into a novel class of anti-rheumatic drug complementary to biological therapies targeting immunity (72). Treatment of AA rats with 2-DG significantly reduces joint swelling, diminishes bone destruction, inhibits synovial cell proliferation and migration, and decreases synovial cell secretion. 2-DG (200 mg/kg) significantly decreases TNF-α, IL-1, and nuclear factor κB ligand (RANKL) levels secreted by synovial cells, while it significantly increases osteoprotegerin (OPG) expression (80). RANKL belongs to the TNF superfamily and constitutes the dominant regulator of osteoclast formation and bone resorption, it binds RANK to activate osteoclasts (91). OPG, an osteoclast inhibitory factor, decreases osteoclast differentiation and activation by inhibiting RANKL-RANK interactions (92). 2-DG reverses CD4 CM-induced FLS activation, decreases the rate of glycolysis, and downregulates H-II, GLUT1, GLUT3, LDHA, PFKFB3, VEGF, and MMP-3 (34). 2-DG, LND, and BrPA are latent agents for the treatment of RA that inhibit HK-II through a variety of modes of action.

## PFK-1/PFKFB3

Analyses of FLSs and ST by RT-qPCR, Western Blot, and IHC have shown increased transcription and expression rates of PFKFB3 in patients with RA compared with the rates in patients with OA. PFKFB1 mRNA expression has not been detected in FLSs, and the expression levels of PFKFB2 and PFKFB4 are similar in the FLSs of patients with RA and OA (93). By contrast, Saeki N’s research demonstrated the mRNA expression levels of Pfkfb1 and Pfkfb3 are up-regulated in murine arthritis tissue-derived synovial macrophages (ADSM) treated with arthritis tissue-derived SF (ADSF)-CM compared to the levels in ADSM treated with ADSM-CM and normal tissue-derived SF (NDSF)-CM (94). PFK15 is a small molecule PFKFB3 inhibitor with potent anti-PFKFB3 activity (95). PFKFB3 inhibition by PFK15 or PFKFB3 siRNA have been used to evaluate the role of PFKFB3 in the pathogenesis of RA. PFKFB3 inhibition reduces the expression of the pro-inflammatory cytokines IL-8 and IL-6, and of the chemokines CCL2 and CXCL10 in RA FLSs; also, PFKFB3 inhibition prevents cell proliferation, migration, and invasion (93). PFK15 inhibits the TNF-α-induced activation of NF-κB, p38, JNK, and ERK MAPK signaling in RA FLSs (93). TNF-α can promote PFKFB3 mRNA expression in primary RA FLSs through STAT3 phosphorylation in combination with OSM (67). In addition, lactate levels in RA FLSs increase after stimulation with TNF-α. However, this increase is inhibited by PFK15 or PFKFB3 siRNA treatment (93). During RA, lactate accumulation regulates the inflammatory immune response (26). To assess the association between lactate and pro-inflammatory cytokines and migration, FLSs were treated with PFK15 for 3 hours and subsequently incubated with 10 mM lactate; results showed that lactate reverses the inhibitory effect of PFKFB3 on the TNF-α-stimulated pro-inflammatory cytokines and chemokines in RA FLSs (93).

Studies have revealed that PFKFB3 silencing inhibits the nuclear translocation of NF-κB-p65 (96), while MAPK encourages PFKFB3 gene transcription and allosteric activation (97, 98). Addition of lactate to PFK15-pretreated RA FLSs reverses the observed reduction in nuclear translocation of p65 and the phosphorylation of IKK and IκBα. The p38, JNK, and ERK activity reductions induced by PFK15 are also reversed by the addition of lactate. This suggests that lactate is involved in the PFKFB3-mediated activation of NF-κB and MAPK in RA FLSs (93). Moreover, selective inhibition of STAT3 using STATTIC, a small molecule JAK/STAT inhibitor (99), in LPS-stimulated RA CD14+ monocytes results in almost complete suppression of the expression of inflammatory markers (such as TNF-α, IL-6, IL-1β, IL-27) and chemokines (such as CXCL10 and CXCL11), and it further diminishes the expression of the PFKFB3, HK-II, and GLUT1 proteins (100). In an animal model of RA, PFK15 treatment reduced the inflammatory cell infiltration and synovial proliferation and reduced the infiltration of pannus into calcified cartilage and bone of CIA mice compared with those of dimethyl sulphoxide (DMSO)-treated mice. The levels of IL-6 in serum and synovium decrease in PFK15-treated CIA as compared with the levels in the DMSO group mice (93).

The immunogenetics of RA suggest that abnormal T cell activation pathways exert a crucial part in the disease onset and/or persistence (101). Determinants of T cell differentiation and survival include antigen recognition, and metabolic mechanisms that provide energy and biosynthetic molecules for the cell building (102). A crucial RA feature of T cells is the transcriptional inhibition of the glycolytic enzyme PFKFB3, resulting in a slow glycolytic flux, reduced ATP and pyruvate production, and reduced extracellular environment acidification (103). Hohensinner PJ et al. summarized this as: in essence, they are “hungry” and energy deprived (104). Glycolytic activation in normal naive CD4+ T cells occurs in response to upregulation of GLUT, which increases glucose uptake and the activity of several rate-limiting enzymes, including PFK-1 (105). The evidence supports the hypothesis that the T cells from RA patients adopt a disparate metabolic program than healthy T cells, which is consistent with autoimmune effector functions dependent on specific energy sensing, energy production, and energy utilization pathways (106). Primary CD4 T cells from RA patients do not metabolize equal amounts of glucose, produce less intracellular ATP, and are inclined to apoptosis as are age-matched control cells (107). This differences are attributed to insufficient induction of PFKFB3. PFKFB3 deficiency occurs in the early stages of the T cell life cycle in patients with RA and has profound metabolic and functional consequences (5). However, this is dissimilar from some other types of autoimmune diseases. For example, CD4+ T cells exert a significant role in pathogenesis of Type 1 Diabetes (T1D) (108). In T1D, CD4+ T cells undergo metabolic reprogramming to the less efficient aerobic glycolysis, similar to that of highly proliferative malignant cells. Inhibition of PFKFB3 *via* PFK15 induces functional and metabolic exhaustion of CD4+ T cells in T1D (109).

To investigate whether RA T cells have an intrinsic glycolysis defect, Zhen Yang et al. measured the expression of 29 glycolytic related genes in activated CD4 T cells from patients with RA and matched controls 72 hours after T cell activation. They found RA T cells were defective in upregulating PFKFB3 compared to controls, with 50% lower transcript levels of PFKFB3 in T cells (107). Mimicking RA T cells, PFKFB3 knockdown decreased PFKFB3-specific transcripts by 50%, encouraging aggressive T cell infiltration with subsequent intense innate and adaptive inflammation, including TNFSF11 expression, indicating recruitment and retention of RANKL+ T cells and tissue production of IL- 1β, IL-6 and TNF (110). The expression of SH3PXD2A, the gene encoding the scaffold protein TKS5, is highly sensitive to metabolic interference. The PFKFB3 inhibitor 3PO (3-[3-pyridinyl]-1-[4-pyridinyl]-2-propen-1-one) on healthy CD4+ T cells mimics the slow glycolytic breakdown of RA T cells, which in turn increases the transcript level of SH3PXD2A (110). The dominating metabolite in inflamed joints is lactate, a decomposition product of glucose produced by metabolically active stromal, endothelial, and invading immune cells (26, 106). Lactate uptake into CD4+ T cells (mediated by the lactate transporter protein SLC5A12) induces remodeling of their effector phenotype, boosting IL-17 production and enhancing fatty acid synthesis *via* nuclear PKM2/STAT3 (111). However, RA T cells do not generate as much ATP and lactate as natural control T cells, they proliferate vigorously instead (112). Three outcome parameters were assessed after knockdown of PFKFB3 in normal T cells: lactate production, intracellular ATP levels, and apoptosis susceptibility. Intracellular ATP production and lactate output were reduced by 25-35%, similar to spontaneous PFKFB3-deficient RA T cells. When PFKFB3 is knocked down, the frequency of Annexin V+ and 7AAD+ cells increases significantly from 5% to 20% (107). Thus, CD4+ T cells in RA produce less lactate and ATP levels than T cells in healthy individuals, suggesting that glycolytic ATP production is the leading source of energy for CD4+ T cells (5).

Mitochondrial and lysosomal abnormalities ultimately lead to the generation of short-lived tissue-invasive effector T cells. This differentiation defect is established on a metabolic platform that shunts glucose from energy production to cell building and motility programs (113). Naive CD4 T cells from patients with RA express an altered pattern of glucose metabolizing enzymes, resulting in slower glycolytic breakdown and increased pentose phosphate pathway (PPP) shunting, favoring anabolic over catabolic reactions (102, 114). A biological consequence of the PPP is the production of NADPH, which is essential for the reduction of oxidized glutathione to glutathione (GSH) and maintains the cellular redox homeostasis (115). What’s more, NADPH protects cells from oxidative toxicity by decreasing ROS levels (116). The disproportionate increase in NADPH (50% higher NADPH levels in RA-derived cells) and reduced oxidized glutathione result in cells that are overconsumed with reactive oxygen species (ROS) and under reductive stress (107). ROS are by-products of oxygen metabolism known for their destructive potential, but contemporary evidence suggests that they play a role as secondary messengers regulating cellular functions through redox-activatable signaling systems (117). Activated and abundant intracellular ROS can regulate cell cycle progression, proliferation efficiency, and naive-to-memory conversion (114). Anti-proliferative measures that induce cell cycle arrest may represent therapeutic approaches against RA (118). ROS-deficient RA T cells are unable to maintain their naive phenotype and bypass the G2/M cell cycle checkpoint to overproliferate (due to surplus reducing equivalents that do not adequately activate the redox-sensitive kinase ATM) (114). CD4+ T cells are able to differentiate into Th1 cells that promote cellular immunity, Th2 cells that support humoral immunity, Th17 cells that promote mucosal immunity, or Treg cells that inhibit the function of effector T cells (26). Th1 and Th17 cells are involved in many autoimmune diseases (119). Insufficient activation of ATM leads to differentiation of T cells towards the Th1 and Th17 lineages, resulting in an excessive inflammatory phenotype (114). The expressions of phosphofructokinase p (PFKp) mRNA and protein are enhanced in RASF in response to ThCM stimulation (79). Inhibition of pro-inflammatory T cell differentiation by correcting reductive stress may suppress synovial inflammation (114).

PFKFB3 deficiency also diminishes the capacity of RA T cells to rely on autophagy as an alternative means of energy and biosynthetic precursor molecules. Knockdown and overexpression of PFKFB3 in TCR-stimulated T cell parental cells. PFKFB3-specific RNA interference inhibits autophagy. In contrast, forced overexpression of PFKFB3 rapidly accelerates autophagic activity (107). PFKFB3 silencing in healthy T cells and overexpression in RA T cells confirm the mechanistic link between glycolytic regulation and autophagy. These studies question the simplistic notion that posits autophagy as the default process for energy production in starving cells. Instead, in T cells, energy production appears to be a coordinated process in which multiple pathways function in parallel (120). To test whether PFKFB3 overexpression can protect RA T cells from autophagy-associated apoptosis, exogenous PFKFB3 reconstitution was combined with treatment with the autophagy inhibitor 3-MA. PFKFB3 overexpression partially rescued RA T cells from 3-MA-induced apoptosis, confirming the upstream position of PFKFB3 in the autophagy regulation stream (107). Moreover, the reduction of cellular ROS inhibits the activation-induced boost of H_2_O_2_ and superoxide. ROS have been associated with enhanced autophagy as a survival strategy. Thus, decreased ROS levels may be an additional mechanism impairing autophagy in RA T cells (57). Essentially, the T cells of patients with RA (even those cells in a naive state) undergo a metabolic reorganization in the presence of insufficient upregulation of the glycolytic enzyme PFKFB3 that leaves them energy deficient, ROS and autophagy deficient, apoptosis sensitive, and senescence prone (107).

**Figure 2:**
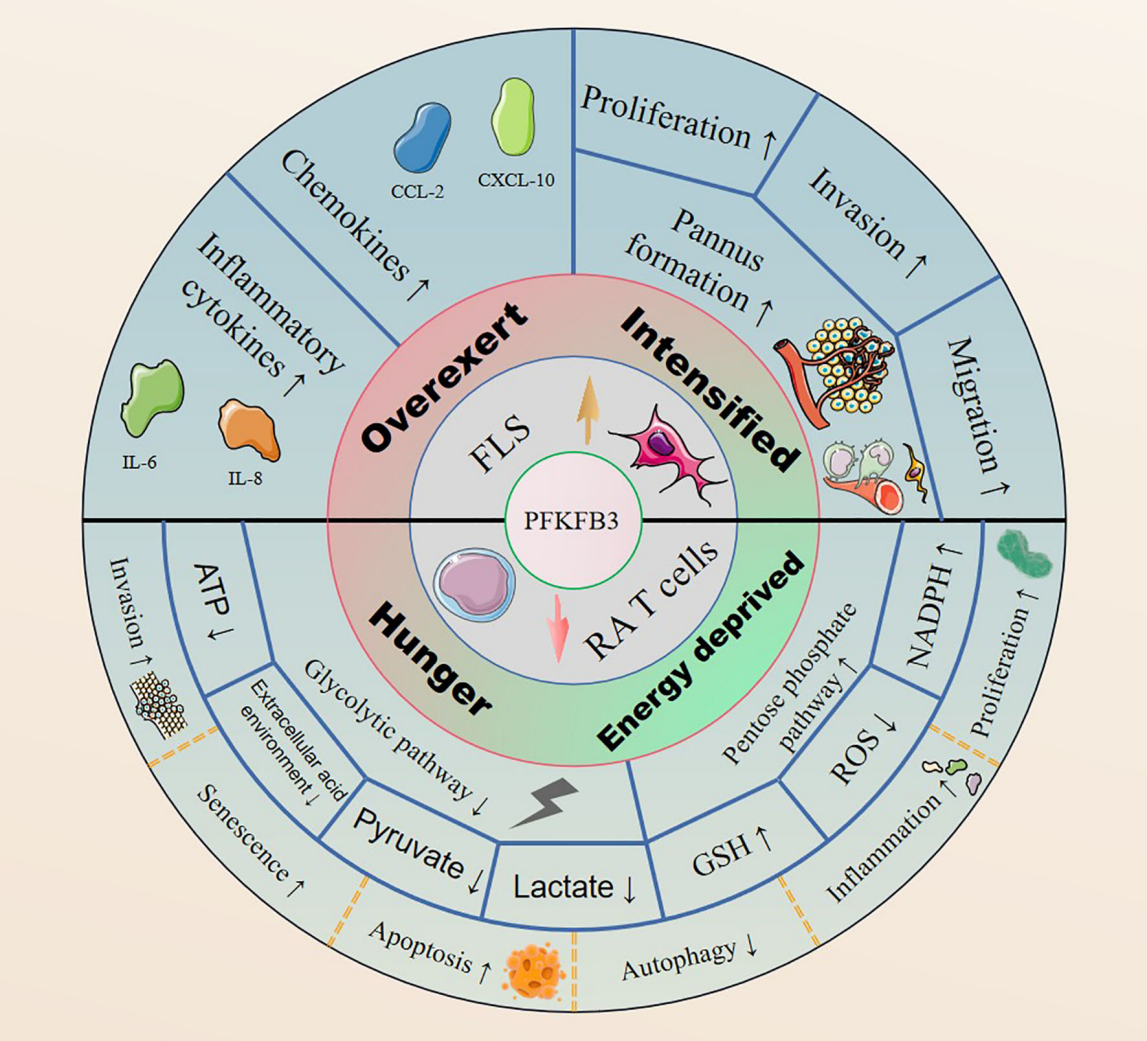
PFKFB3 is highly expressed in RA FLSs, it upregulates the expression of inflammatory cytokines and chemokines, and facilitates cell proliferation, invasion and migration by promoting pannus formation. In contrast, PFKFB3 is expressed at low levels in RA T cells, generating an RA pathological phenotype with an inhibited glycolytic pathway and enhanced PPP shunting.

The expression levels of PFKFB3 can vary in different tissues. PFKFB3’s expression in the normal human body is in a homeostatic state that gets disrupted by either overexpression in FLSs or underexpression in RA T cells (**Figure 2**). By contrast, inhibition of PFKFB3 expression in RA FLSs may effectively improve RA symptoms by inhibiting glycolysis (93). Forced overexpression of PFKFB3 in RA T cells restores the glycolytic flux and protects cells from excessive apoptosis (107). Therefore, controlling the glycolytic pathway by targeting PFKFB3 to treat RA may be possible, but the choice of therapeutic modality and the dosages need to be further investigating.

## PKM2

A MALDI-TOF-MS analysis of 1633 and 1603 protein spots in the synovial FLSs of patients with RA and controls (respectively) showed that the expression of PKM1/M2 protein was more than 3-fold higher in RA FLSs than in control FLSs. In addition, a Western blot assay demonstrated that the PKM2 expression results were consistent with the proteomic analysis (121). PK expression was high in RA synovial cells, indicating that RA STs present increased glycolytic activity (122). Phosphorylated PKM2 (p-PKM2) expression is increased in FLSs and the ST of patients with RA (123). PKM2 expression is higher in the lining and sublining layer, and vascular system of the RA ST than in the OA ST (124). siRNA transfection of PKM2 results in decreased expression of PKM2 and p-PKM2, which in turn results in decreased migration, invasion, expression of inflammatory factors (such as IL-1β, IL-6, and IL-8), glucose uptake and lactate secretion, and expression of LDHA, PDK1, and GLUT1 (123). Studies have described an altered glucose metabolism in RA. Pkm2 has been found overexpressed in ED1-positive macrophages (Mφ) in the spleen and ST of pristane-induced arthritis (PIA) rats by immunofluorescence, Western blots, and RT-qPCR; the overexpressed Pkm2 promotes Mφ activation *via* Stat1 signaling (125). Patient-derived Mø created higher levels of ATP with an interesting RA Mø hierarchy (126). Glycolysis is also upregulated in RA patient-derived Mφ, as are rate-limiting enzymes such as PKM2, PFKFB3, and HK-II, and GLUT1 and GLUT3 (127). GSK-3β inhibition promotes mitochondrial activity, and it enhances ATP synthesis and ROS release in RA patient-derived Mφ. This metabolic constellation leads to a cytoplasmic-to-nucleus translocation of PKM2 with functional consequences that include PKM2-dependent activation of STAT3, which promotes the production and secretion of pro-inflammatory cytokines such as IL-6 and IL-1 (128). Toll-like receptor 2 (TLR2)-activation regulates bioenergetic profile changes in primary RASFs consisting of PKM2 nuclear translocation, mitochondrial respiration and ATP synthesis reductions, and glycolysis increases (124). Inflammatory tissue lactate levels in CD4+ T cells induce IL17 expression through nuclear PKM2 and fatty acid synthesis-mediated STAT3 phosphorylation (111). PKM2 is significantly upregulated in activated CD4+ T cells and is required for pro-inflammatory Th17 and Th1 cell differentiation (129). The increase in aerobic glycolysis mediated by activated T helper cells pushes SFs towards an inflammatory phenotype. In response to ThCM stimulation, the mRNA and protein expressions of PKM2 and lactate dehydrogenase A (LDH-A) in RASFs get induced (79).

PDPN is highly expressed in cadherin-11-positive cells throughout the RA synovial lining layer. This expression is most pronounced in sections with hyperplasia and high matrix metalloproteinase-9 (MMP-9) expression, where it coincides with upregulation of α-smooth muscle actin (α-sma) (130). CD45-PDPN+ FLS cells from the K/BxN mouse serum transfer model of arthritis and enriched in culture show significantly higher expressions of PKM2 mRNA, GLUT1, LDHA and ENO1 than CD45-PDPN- cells (33). A comparative transcriptomic analysis has shown PMK2 upregulation in human IgG-trained monocytes compared to its expression in controls (40). SUMOylation is an important modification with a regulatory role in cellular responses to various types of stress including osmotic, hypoxic and oxidative stress (131). SUMOylation occurs through a series of stress-induced biochemical responses (132). Oxidative stress is a contributing factor in the pathogenesis of RA and influences the development of the RA process through multiple pathways (133, 134). RA as an autoimmune disease and SUMOylation is a novel pathway involving its phenotypic differences (135). Wang C et al. found increased expression of SUMO-activating enzyme subunit 1 (SAE1) and ubiquitin like modifier activating enzyme 2 (UBA2) in FLSs and ST of patients with RA, where SAE1/UBA2 regulated the glycolytic pathway and biological functions of the RA FLSs through SUMOylation-mediated PKM2 phosphorylation (123). For instance, in lung cancer cells, SUMO1 promotes PKM2-dependent glycolysis. SUMO1 modification of PKM2 has been proposed as a therapeutic target against lung cancer (136). In RA FLSs, treatment by siRNA knockdown of SAE1 or UBA2 with GA, an inhibitor of SAE1/UBA2-mediated SUMOylation, resulted in reduced glycolysis, inflammatory and aggressive phenotype (123).

In an experiment where Dark Agouti (DA) rats were treated intraperitoneally with either shikonin or an RNA-interfering plasmid of PKM2 and a negative control plasmid, respectively (125). Shikonin is a specific PKM2 inhibitor that inhibits cellular aerobic glycolysis and cell proliferation by reducing PKM2 activity (137, 138). Pkm2 intervention reduced the severity of PIA, including macroscopic arthritis scores, perimeter changes of midpaw, synovitis, and bone and cartilage destruction, and reduced ST in rat ED1 and p-Stat1-positive cell populations (125). Inhibition of PKM2 reduces phosphorylation levels of STAT1 and STAT3 and inhibits the transcription of downstream genes regulating pro-inflammatory cytokines, thereby alleviating experimental arthritis (129). In classically activated rat and mouse Mφ, silencing Pkm2 by RNA interference results in less production of TNF-α and Il-1β *via* Stat1 signaling (125). Blocking ICOS (a member of the CD28 superfamily) signaling during TH cell and B cell co-culture successfully inhibits the upregulation of the key rate-limiting glycolytic enzymes PKM2, HK-II, PFKFB3, and PFKL in TH cells (71).

## Future Direction

Metabolic disorders and changes in the intracellular levels of specific metabolites are associated with an inflammatory phenotype of immune cells that has been associated with autoimmune diseases such as systemic lupus erythematosus, RA, multiple sclerosis, and diabetes mellitus (139). Targeting key metabolism players (such as mTOR by rapamycin, HKs by 2-DG, and AMP-activated protein kinase by metformin) can improve autoimmune inflammation (139–142). Tofacitinib is the first disease-modifying anti-rheumatic drug (DMARD) approved for the treatment of RA (143). Its efficacy and safety profile as an oral Janus kinase inhibitor for the treatment of RA is promising (144). Tofacitinib inhibits the mRNA expression of HK-II, GLUT1, PFKFB3, 3′-phosphoinositide-dependent protein kinase 1 (PDK-1), and GSK-3α in RA whole-tissue synovial organotypic explants *ex vivo*, as well as the expression of the pro-inflammatory cytokines IL-6, IL-8, and IL-1β, the key adhesion molecule soluble intercellular adhesion molecule 1 (sICAM), and the growth factors TIE-2 and vascular endothelial growth factor (VEGF) (145). The successful application of DMARDs has provided insights for the potentially effective development of therapeutic agents for RA based on modulators of key glycolysis rate-limiting enzymes (**Table 1**).

**Table 1:** Some common molecular inhibitors of glycolysis rate-limiting enzymes.

Molecules	Molecular Formula	Molecular Weight	2D Structure	Notes	References
**HK-II**					
3-BrPA	C_3_H_3_BrO_3_	166.96	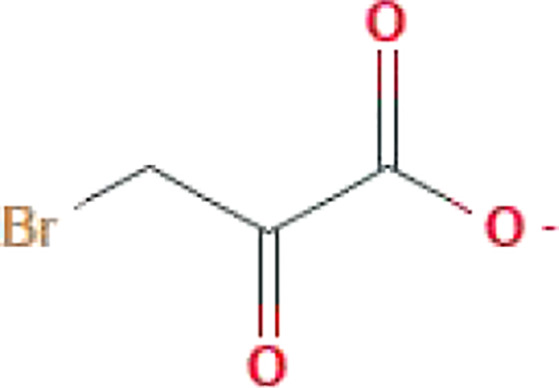	The structural similarity to lactate	(146)
2-DG	C_6_H_12_O_5_	164.16	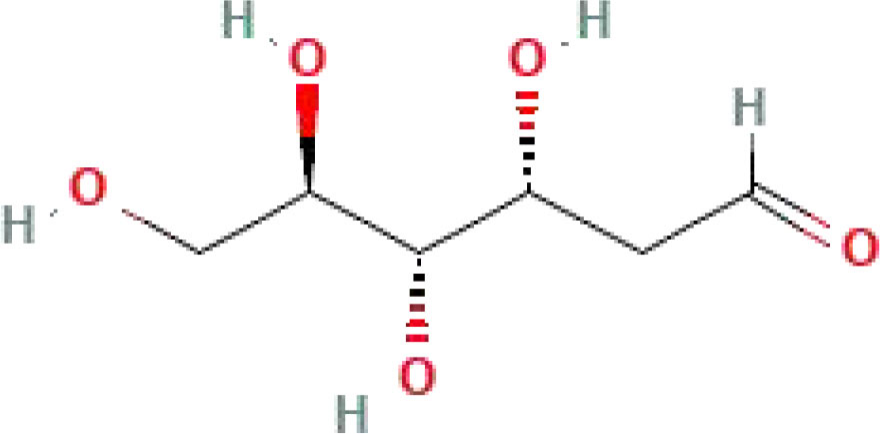	Noncompetitive antagonist	(75, 85)
Lonidamine	C_15_H_10_Cl_2_N_2_O_2_	321.2	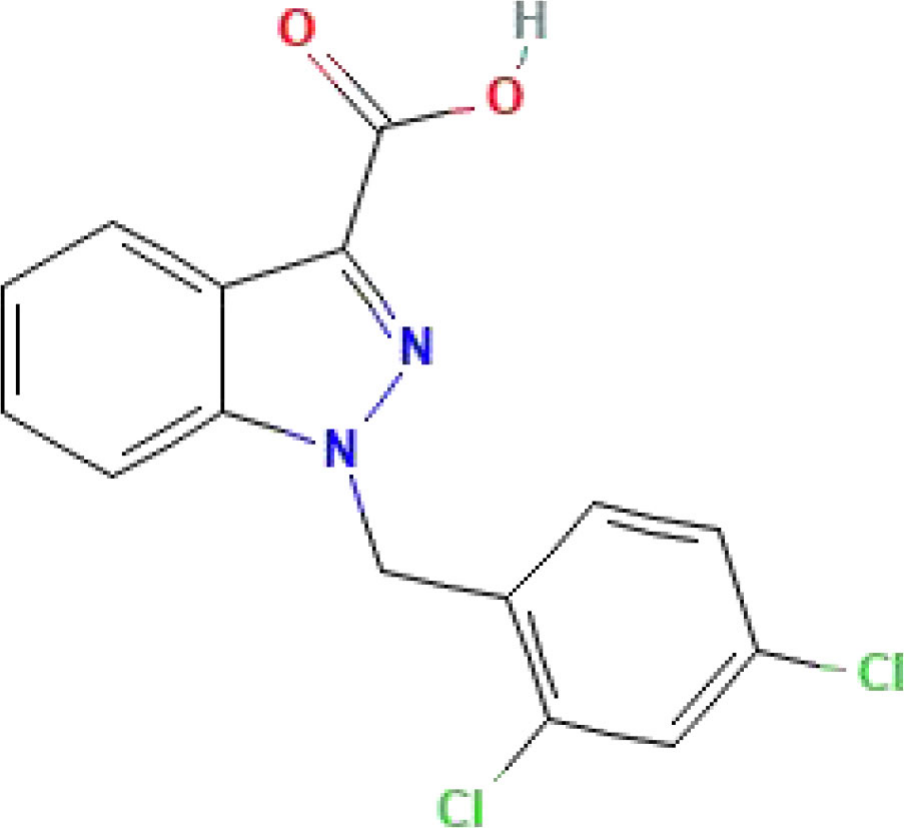		(147)
Metformin	C_4_H_11_N_5_	129.16	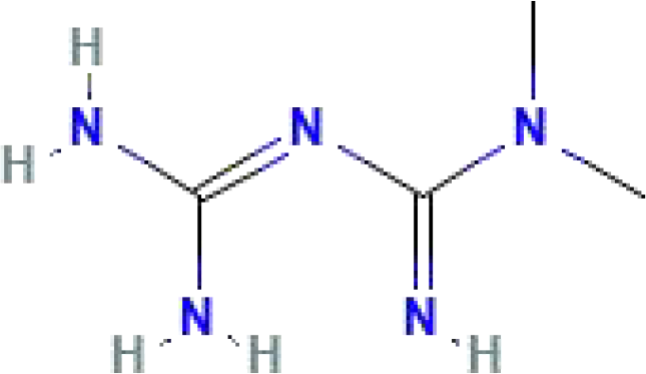	Metformin could offset the increased compensatory effect in oxidative respiration in HK-II silenced cells	(148, 149)
Benserazide	C_10_H_15_N_3_O_5_	257.24	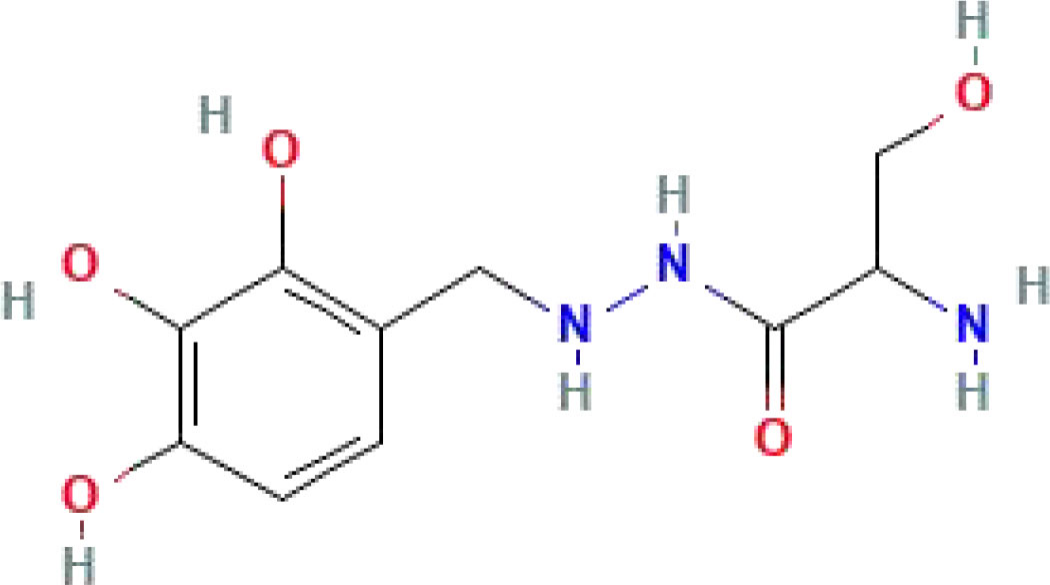		(150)
Costunolide	C_15_H_20_O_2_	232.32	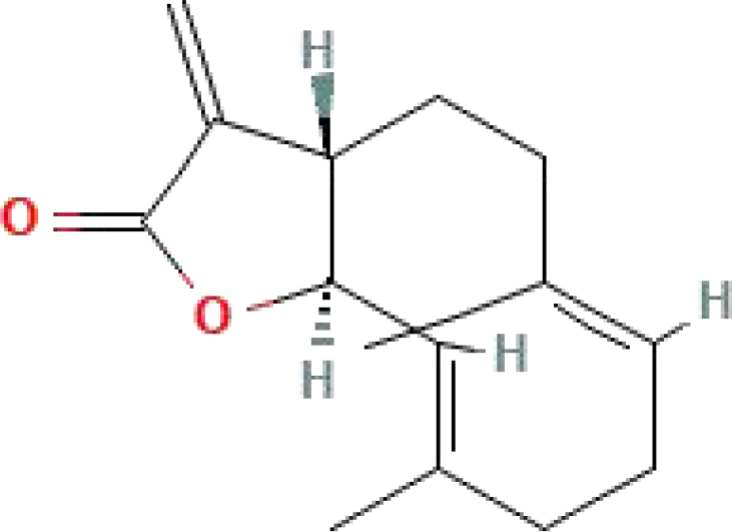	A well-characterized sesquiterpene lactone compound	(151)
**PFKFB3**					
PFK-15	C_17_H_12_N_2_O	260.29	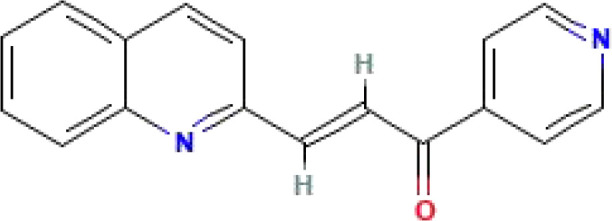		(47, 152)
PFK-158	C_18_H_11_F_3_N_2_O	328.3	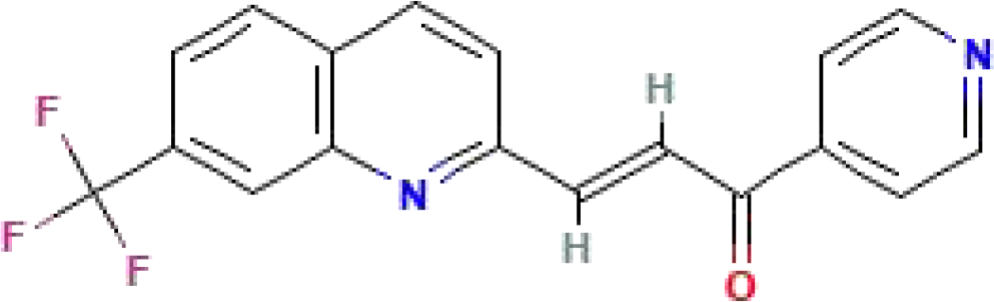	The first-in-man PFKFB3 inhibitor to be evaluated in a phase I clinical trial	(47, 153, 154)
3PO	C_13_H_10_N_2_O	210.23	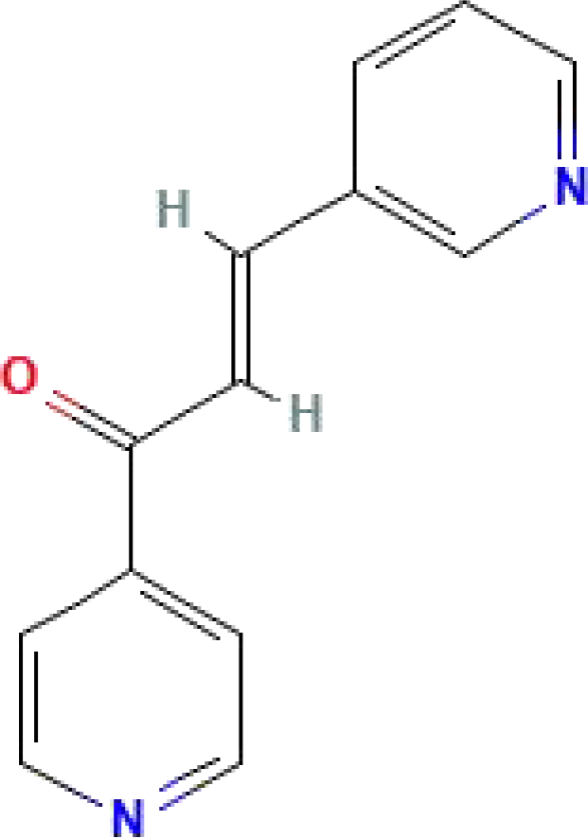	The mechanism of action is controversial	(155–157)
KAN0438757	C_21_H_18_FNO_7_S	447.4	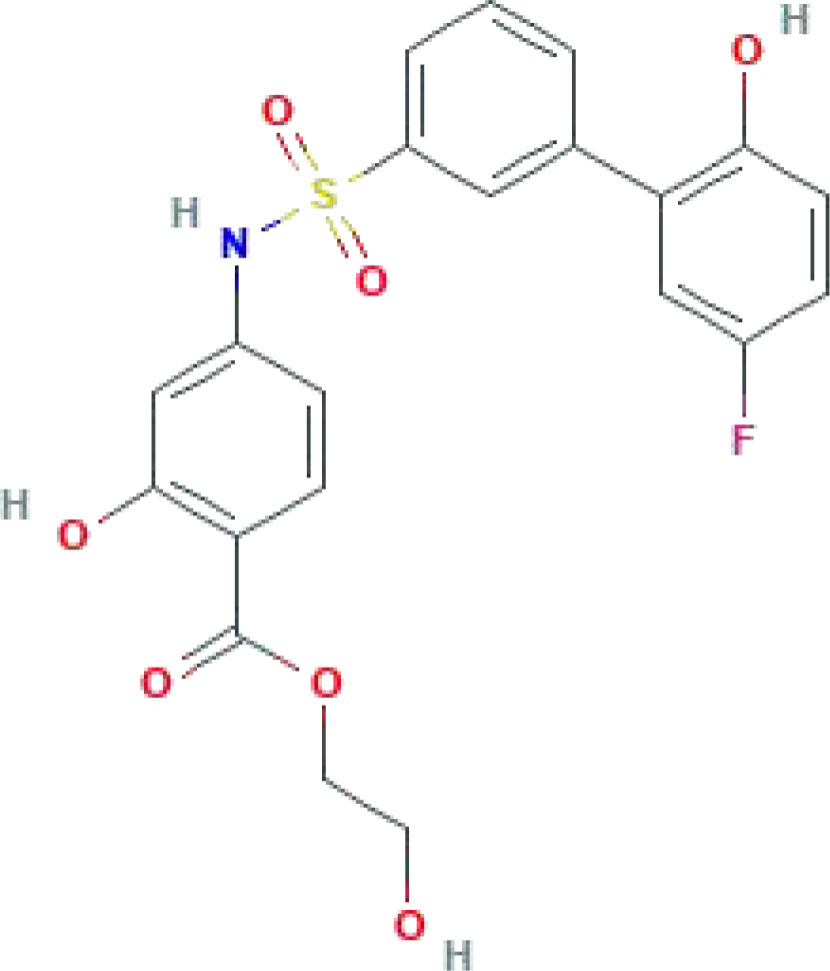		(158, 159)
**PKM2**					
Shikonin	C_16_H_16_O_5_	288.29	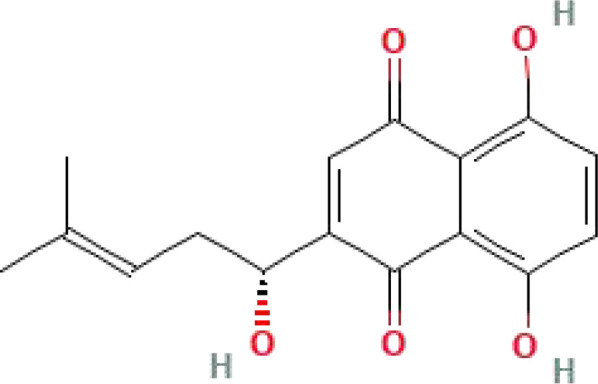	Currently the most commonly used PKM2 inhibitors	(47, 160)
Alkannin	C_16_H_16_O_5_	288.29	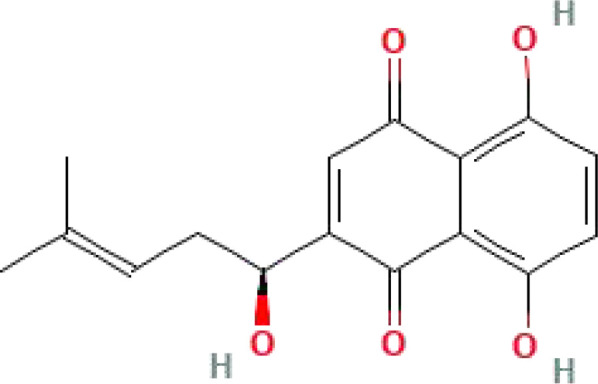	The enantiomeric isomer of shikonin	(161)
Gliotoxin	C_13_H_14_N_2_O_4_S_2_	326.4	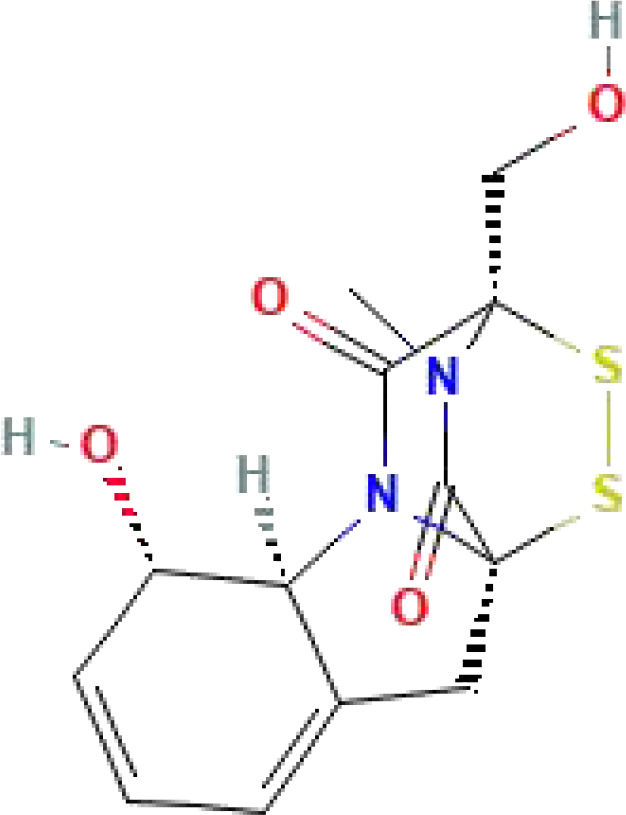	IC_50_ = 22.64 μM	(162)
Benserazide	C_10_H_15_N_3_O_5_	257.24	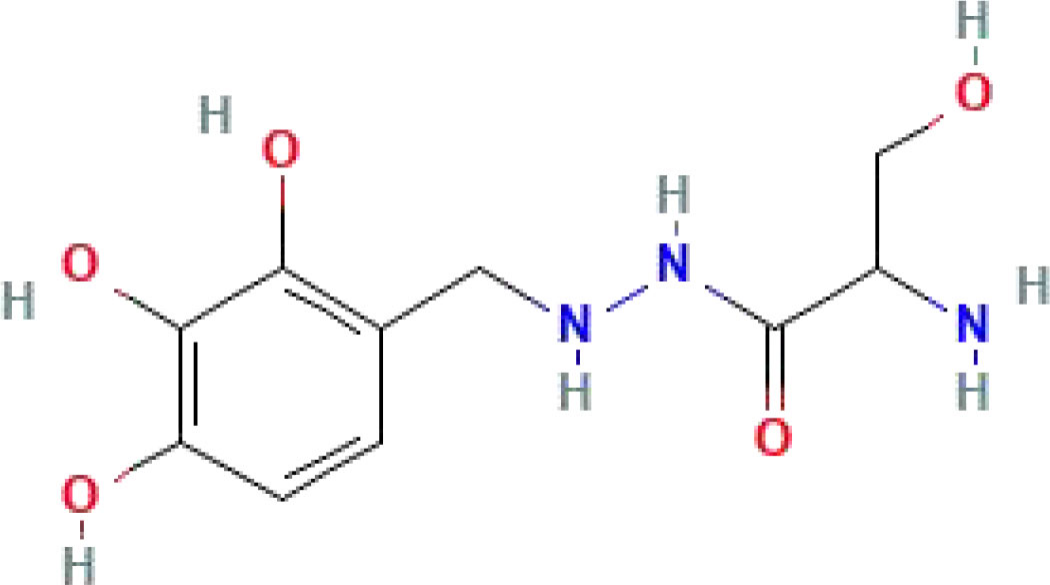		(163)

HK-II blockade represents a novel therapeutic strategy for RA that ameliorates inflammation and cartilage damage in a K/BxN arthritis model (164). 2-DG, a non-competitive inhibitor of HKs, reverses LPS-induced enhancement of glycolytic activity and inhibits the expression of vital inflammatory cytokines (IL1-β, IL6) and matrix metalloproteinases (MMP-1, MMP-3) in the RA pathogenesis (75). Targeting the cAMP response element binding protein (CREB) is a potential idea to treat RA (165). Aberrant cAMP/CREB signaling has a crucial role in inducing imbalance in Mø polarization (166), and in promoting osteoclast differentiation in RA (167). 2-DG inhibits phosphorylation of the LPS-enhanced transcription factor CREB (75). A preventive glycolytic pathway inhibition with 2-DG significantly limits antibody-mediated pathology in the K/BxN model of RA, most likely *via* its effects on Tfh cells. Metformin2 has been shown to inhibit HK-II activity and induce HK-II dissociation from mitochondria, but it has no inhibitory effects on HK-IV (47, 148). However, the combination of metformin 2 and 2-DG has little beneficial effects in the course of RA treatment. The addition of metformin 2 inhibits the compensatory switch to the oxidation of other substrates, limiting the efficacy of the glycolytic pathway inhibition (81). 3-BrPA is a halogenated analogue of pyruvate known for forty years as an alkylating agent that reacts with the thiol groups of many proteins. It is based on impairing the energy metabolism of tumor cells by inhibiting enzymes in the glycolysis, HK-II, glyceraldehyde 3-phosphate dehydrogenase, phosphoglycerate kinase, and oxidative phosphorylation, succinate dehydrogenase (168). 3-BrPA effectively ameliorates RA-related symptoms of CIA due to the overlap between ICOS signaling, phosphoinositide 3-kinase (PI3K) signaling and glucose metabolism (169). Addition of 3-BrPA to block glycolysis in Th cell-stimulated SF significantly reduces lactate production and the ratio of glycolysis to oxidative glucose metabolism. In addition, 3-BrPA suppresses the pro-inflammatory phenotype by strongly reducing the secretion of IL-6 and MMP-3 in ThCM-stimulated SF (79). In conclusion, overexpression of HK2 in FLS is closely related to inflammatory phenotypes of RA, and therapeutic interventions targeting HK2 by inhibitors such as 2-DG and 3-BrPA are important for the future DMARDs exploitation. The discovery of novel inhibitors with their specific mechanisms of action is of biomedical importance to explore optimal therapies for the prevention and treatment of RA.

The *in vivo* effects of PFK15 (a selective PFKFB3 inhibitor) on RA synovial inflammation and joint destruction have been evaluated in CIA mice: Intraperitoneal injection of PFK15 reduces the increase in clinical scores compared to DMSO treatment (93). In RASFs, blockade of glycolysis by 3PO, another PFKFB3 inhibitor (47), reverses TLR2-induced pro-inflammatory mechanisms, including invasion, migration, and secretion of IL-6, IL-8, monocyte chemoattractant protein-1 (MCP-1), normal T-cell expressed and secreted (RANTES), and growth-regulated oncogene alpha (GRO-α) (124). Attempts to modify the pathological process of RA by targeting PFKFB3 are attractive, but the diverse expression of PFKFB3 in different tissues of RA poses a challenge to such studies. Treatment of RA with PFKFB3 inhibitors such as PFK15 or 3PO may reduce pathological phenotypes of RA, but whether this process further aggravates the intrinsic PFKFB3 defect in RA CD T cells, further studies are needed to gain insight into the detailed mechanism of action.

Daurinol is a novel topoisomerase II inhibitor isolated from the traditional medicinal plant Haplophyllum dauricum (170). Real-time PCR analysis has shown that daurinol treatment of murine CD4+ T cells cultured under Th17-polarizing conditions downregulates genes encoding for various molecules involved in aerobic glycolysis, such as HK-II, PKM, GLUT1, monocarboxylic acid transporter member 4 (MCT4), GPI, triosephosphate isomerase (TPI), Eno1, compared to vehicle-treated cells. Moreover, treatment with daurinol reduces the development of inflammatory arthritis in a dose-dependent manner and inhibits osteoclastogenesis *in vitro* and *in vivo* (171).

Studies have found no significant changes in kidney, serum creatinine, liver, ALT and AST, or serum glucose in mice treated with PFK15. Additionally, there were no significant histopathological alterations in the liver or kidneys removed from PFK15-treated mice compared to those in DMSO-treated groups. These data demonstrate the safety of PFK15 treatment in CIA mice (93). The viability of SFs was not affected by the 3-BrPa concentrations used for experiments (79). However, other studies have reported bloody ascites, abdominal distention, and organ cirrhosis in some rats treated with 3-BrPA (172). In rabbits, selective intra-arterial injection of 25 mM 3-BrPA can cause considerable toxicity in the liver and in the gastrointestinal system, and this dose-dependent toxicity can lead to death at high doses (173). Therefore, toxicological studies during the development of these drugs are needed to be able to direct necessary drug modifications while maintaining their efficacy and minimizing adverse effects.

## Conclusion

Studies have emphasized the association between inflammatory tissue damage (induced by cytokines, chemokines and ROS) and RA pathogenesis. However, the overwhelming majority of life activities are energy-dependent, and metabolism changes are usually prominent disease effectors. “Pull one hair and the whole body is affected”; therefore, exploring the pathogenesis of RA from a metabolic energy point of view is a valid approach. Our understanding of the role of glucose metabolism in RA is incomplete, but studies on glycolytic rate-limiting enzymes have greatly expanded our understanding of the energy metabolism interaction network. The glycolysis rate-limiting enzymes HK-II, PFK-1/PFKFB3, and PKM2 can act as regulators of inflammatory factors, chemokines, and growth factors, which in turn play important roles in the development of RA. Studies on HK-II, PFK-1/PFKFB3 and PKM2 in the pathogenesis of RA have facilitated the development of new DMARDs targeting rate-limiting enzymes of the glycolytic pathway to implement new anti-rheumatic therapies. In conclusion, verifying whether glycolysis is important in the pathogenesis of RA and exploring the mechanisms of key glycolytic pathway rate-limiting enzymes in RA is a valid and promising avenue.

## Author Contributions

JZ and JT have contributed equally to this work and share first authorship. JZ, JT, and PS contributed to conception and design. JZ and JT wrote the manuscript and figures. ML and ZZ collected the data and designed the figures. YL, HT, EL, BG, and TL performed literature search, and provided valuable comments. All authors contributed to the article and approved the submitted version.

## Funding

The study was financially supported by the Science and Technology Development Program of Jilin Province (Nos. 20190304124YY, 20200201429JC), the National Natural Science Foundation of China (No. 81903273), the Department of Finance of Jilin Province (No. 2020SCZ63), and the Bethune project of Jilin University (No. 2020B36).

## Conflict of Interest

The authors declare that the research was conducted in the absence of any commercial or financial relationships that could be construed as a potential conflict of interest.

## Publisher’s Note

All claims expressed in this article are solely those of the authors and do not necessarily represent those of their affiliated organizations, or those of the publisher, the editors and the reviewers. Any product that may be evaluated in this article, or claim that may be made by its manufacturer, is not guaranteed or endorsed by the publisher.
